# Neutrophils from severe asthmatic patients induce epithelial to mesenchymal transition in healthy bronchial epithelial cells

**DOI:** 10.1186/s12931-019-1186-8

**Published:** 2019-10-29

**Authors:** Alexandre Haddad, Mellissa Gaudet, Maria Plesa, Zoulfia Allakhverdi, Andrea K. Mogas, Severine Audusseau, Carolyn J. Baglole, David H. Eidelman, Ronald Olivenstein, Mara S. Ludwig, Qutayba Hamid

**Affiliations:** 1 Translational Research in Respiratory Diseases, Meakins-Christie Laboratories, Research Institute of the McGill University Health Centre, 1001 Boulevard Décarie, Montréal, QC H4A 3J1 Canada; 2 Faculty of Medicine, McGill University, Montréal, Canada; 3 College of Medicine, University of Sharjah, Sharjah, United Arab Emirates

**Keywords:** Epithelial-mesenchymal transition, Airway remodeling, Severe asthma, Neutrophils, TGF-β1

## Abstract

**Background:**

Asthma is a heterogenous disease characterized by chronic inflammation and airway remodeling. An increase in the severity of airway remodeling is associated with a more severe form of asthma. There is increasing interest in the epithelial to mesenchymal transition process and mechanisms involved in the differentiation and repair of the airway epithelium, especially as they apply to severe asthma. Growing evidence suggests that Epithelial-Mesenchymal transition (EMT) could contribute to airway remodeling and fibrosis in asthma. Severe asthmatic patients with remodeled airways have a neutrophil driven inflammation. Neutrophils are an important source of TGF-β1, which plays a role in recruitment and activation of inflammatory cells, extracellular matrix (ECM) production and fibrosis development, and is a potent inducer of EMT.

**Objective:**

As there is little data examining the contribution of neutrophils and/or their mediators to the induction of EMT in airway epithelial cells, the objective of this study was to better understand the potential role of neutrophils in severe asthma in regards to EMT.

**Methods:**

We used an in vitro system to investigate the neutrophil-epithelial cell interaction. We obtained peripheral blood neutrophils from severe asthmatic patients and control subjects and examined for their ability to induce EMT in primary airway epithelial cells.

**Results:**

Our data indicate that neutrophils from severe asthmatic patients induce changes in morphology and EMT marker expression in bronchial epithelial cells consistent with the EMT process when co-cultured. TGF-β1 levels in the culture medium of severe asthmatic patients were increased compared to that from co-cultures of non-asthmatic neutrophils and epithelial cells.

**Conclusions and clinical relevance:**

As an inducer of EMT and an important source of TGF-β1, neutrophils may play a significant role in the development of airway remodeling and fibrosis in severe asthmatic airways.

## Introduction

Asthma, a complex heterogeneous disorder, with a broad spectrum of phenotypes, continues to increase globally and remains a major illness in terms of morbidity, mortality and cost (1). Asthma is classically considered an allergic, T-helper type 2 (T_H_2) cell driven inflammation, characterized by eosinophilic infiltration of the airway. Research has focused on the role of T_H_2 cells and cytokines (IL-4, IL-5, and IL-13) in contributing to asthma pathogenesis (2). However, subgroups of asthmatic patients with a more severe form of the disease exhibit refractory symptoms, with little to no eosinophil infiltration of the airway. The airway inflammation in severe asthma, which differs from mild or moderate persistent asthma, is characterized by the influx of neutrophils in sputum, bronchoalveolar lavage fluid (BALF) and biopsy specimens, with or without eosinophilia (1, 3–5). Airway neutrophilia has been shown to be associated with more severe airflow obstruction, lower lung function and thicker airway walls (6–8).

Airway remodeling is an important pathologic feature of asthma, and occurs in both the central and peripheral airways (9). The airway structural changes include injury and shedding of airway epithelium, enlargement of goblet cell and submucosal glands, increased myofibroblast number, subepithelial fibrosis, increased airway smooth muscle (ASM) mass and neovascularization (10–14). These changes contribute to the thickening of airway walls, increased mucus secretion and airway hyper-responsiveness and thereby lead to airway narrowing and airflow obstruction. The extent of airway remodeling is positively correlated with disease severity.

These changes, especially sub-epithelial fibrosis, may play an important role in disease pathogenesis and physiologic dysregulation. Chronic inflammation is believed to be the major contributor to airway remodeling in asthma via ongoing activation of inflammatory cells such as eosinophils, mast cells, T-cells and neutrophils. Substantial effort has been dedicated to trying to better understand and describe the mechanisms by which inflammation leads to airway remodeling. One mechanism which may play a significant role in airway remodeling is epithelial-mesenchymal transition (EMT).

During EMT, epithelial cells lose their apical-basolateral polarity and cell-cell adhesions and acquire a mesenchymal phenotype with an enhanced migratory capacity (15) and the decreased expression of E-cadherin would be expected in these conditions (16). Epithelial cells undergoing EMT reorganize their cytoskeletons and transition into a spindle-like morphology. They have increased mesenchymal protein expression such as N-cadherin, α-smooth muscle actin and vimentin (17–20). EMT can be classified into three functionally distinct categories (21). Type II EMT is relevant in asthma and is involved in tissue repair and wound closure, via generation of a pool of mesenchymal cells that is required for tissue regeneration (22). Type II EMT can persist beyond the inflammatory process and lead to pathological fibrosis. Recently, the bronchial epithelium has been studied as a source of fibroblasts and myofibroblasts which are important players in airway remodeling in asthma (23). Chronic inflammation may lead to uncontrolled tissue repair by Type II EMT consequent to repeated damage of the epithelium by allergens, infections, allogenicity, cigarette smoke, etc. This can result in excessive production of ECM proteins by fibroblasts and myofibroblasts, ultimately leading to tissue fibrosis and remodeling. There is increasing evidence for the involvement of EMT in asthma, in vitro and in murine models (4, 23–26).

Transforming growth factor-β1 (TGF-β1) is a potent and well described inducer of EMT, and a profibrotic growth factor with immunoregulatory properties. Eosinophilic derived TGF-β1 has been linked to fibrosis in asthmatic airways, and is a known pro-fibrotic factor in the airway wall of allergic (predominantly eosinophilic) asthmatics (27). Increased levels and expression of TGF-β1 have been shown in BALF and in bronchial biopsies of asthmatics with a more severe form of disease (27–30). Evidence from patients with asthma suggests that TGF-β1 protein and mRNA correlates with the thickness of subepithelial basement membrane (29, 30). In vitro studies have also shown that fibroblast to myofibroblast and epithelial to mesenchymal transitions can be induced by TGF-β1 (31). Targeted expression of TGF- β1 in newborn and adult rat lungs induces a dramatic fibrotic response with minimal inflammation. This response is characterized by extensive deposition of the extracellular matrix proteins collagen, fibronectin, and elastin, and by emergence of cells with the myofibroblast phenotype.

Neutrophils are central players in the inflammatory process present in asthma and have the capacity to produce TGF-β1. Airway neutrophilia is associated with more severe airflow obstruction, lower lung function and thicker airway walls (6–8). Wenzel et al. showed that neutrophil numbers are increased in BALF collected from severe asthmatic patients but not in mild asthmatic or moderate asthmatic patients (32). The relationship between airflow obstruction and airway neutrophilia has also been reported by Shaw et al. in a group of 1100 asthma patients. They found that both neutrophilia and eosinophilia were associated with low prebronchodilator FEV1, but only neutrophilia was associated with low post-bronchodilator FEV1 (33).

These studies demonstrate that airway neutrophilia is a characteristic of severe asthma and suggest that neutrophils have a significant role in the airway narrowing of asthmatic patients. In addition to producing TNF-α, IL-1β, IL-3, IL-6, IL-8, and GM-CSF (34), neutrophils are known to produce TGF-β1 (35). Interestingly, TGF-β1 can both recruit and activate neutrophils (36). It can also prolong neutrophil survival (37, 38). Chu et al. have reported that airway and blood neutrophils from both asthmatic and normal subjects can express and release TGF-β1, and that culture supernatants from asthmatic peripheral blood neutrophils released significantly higher levels of TGF-β1 than those from normal control subjects (39). Although these studies document accumulations of neutrophils in the airway walls of severe asthmatics, they also raise questions about the role these cells play in the process of airway remodeling. This has led us to consider the hypothesis that neutrophils induce EMT process via TGF-β1, and thereby contribute to the pathophysiology of severe asthma. This study will investigate the neutrophil-epithelial cell interaction in the context of EMT and asthma using an in vitro system. Our results lead us to conclude that neutrophils may play a significant role in the development of airway remodeling and fibrosis in severe asthmatic airways as an inducer of EMT and an important source of TGF-β1.

## Methods

### Human bronchial epithelial cell isolation

Normal primary human bronchial epithelial cells (NHBE) were purchased from LONZA (Walkersville, USA) or directly isolated from healthy human airways post-mortem. In the latter case, the lungs were kept on ice and processed within 24–48 h after surgery. Fresh human bronchial segments were obtained from the right middle lobe and cut longitudinally. “Strips” of epithelial cells were then identified using a light microscope and were gently detached from the bronchial segments. These tissue explants were allowed to adhere in a petri dish using the PneumaCult medium complemented with Penicillin (10,000 Unit/ml) Streptomycin (10,000 μg/ml) and Amphotericin B (25 μg/ml) and left to incubate at 37 °C. Within 1–3 weeks, cells started growing from the explants and populating the petri dish. No differences in the outcome of the experiments were observed using purchased NHBE vs bronchial epithelial cells isolated post-mortem.

### Human bronchial epithelial cell culture and treatment

Bronchial epithelial cells were cultured in culture flasks using the bronchial epithelial cell culture medium PneumaCult (Stemcell Technologies, Vancouver, CA) complemented as described above. When a confluence of 75% was reached, the flask was washed with sterile PBS and the cells were detached by trypsinization. Cell counts were performed manually using a haemocytometer (a 0.4% Trypan blue solution was mixed with an equal volume of cell containing media), or automatically using an automated cell counter (60 μm tip). Both methods yielded similar results. Cells were seeded in 12 well plates at a density of 50,000 cells per well, or in 6 well plates at 100000 cells per well. The cells were then incubated with either fresh media containing TGF-β1 (10 ng/ml) (Bio-Rad, CA), 0.1–1.0 × 10^6^ neutrophils/ml, or exposed to neutrophil-conditioned media (derived from 0.1–1.0 × 10^6^ neutrophils/ml) for 48 h, as below.

### Neutrophil isolation

Peripheral venous blood was obtained from healthy non-asthmatic and severe asthmatic subjects. Severe asthmatic individuals had a forced expiratory volume at 1 s (FEV1) lower than 80% of vital capacity (VC) and poor symptom control (asthma control test scores < 20 or asthma control questionnaire score > 1.5) despite high doses of ICS or OCS. These patients had also experienced one or more severe exacerbations requiring hospitalization in the past year. Patient characteristics are shown in Table 1. Neutrophils were isolated from peripheral venous blood by column-free immunomagnetic separation using an EasySep™ Direct human neutrophil isolation Kit (Stemcell Technologies, Vancouver, CA.) The cells were suspended and cultured in enriched Roswell Park Memorial Institute 1640 medium (RPMI) (Life Technologies, Carlsbad, USA) without serum.

**Table 1:** Clinical characteristics of subjects included in the study

	Asthmatics	Non-asthmatics
Subjects (n)	15	4
*Severity*		
Mild	1/15	
Severe	14/15	
Female / Male (n)	7/8	3/1
Age	51.8 ± 11	47.5 ± 15
*Ethnic group (n)*		
Caucasian	11	4
North indian/pakistan	1	
African descendant	1	
Asian	1	
Weight (kg)	74.83 ± 21.6	67.25 ± 13.4
Height (m)	1.66 ± 0.09	1.64 ± 0.03
FEV1 (l)	2.5 ± 0.77	3.07 ± 0.45
FEV1 (%)	79.7 ± 22.2	102 ± 15.2
FEV/FVC	0.70 ± 0.12	0.83 ± 0.008

### Neutrophil-epithelial cell co-cultures

Bronchial epithelial cells were cultured in 6- or 12-well plates until 60–70% cell confluence. Human neutrophils were then added to the culture (up to 1 × 10^6^ neutrophils for 12-well plates). In preliminary experiments we determined that after 48 h, only 34% of neutrophils were still viable, whereas after 24 h, 65% of the neutrophils were still viable. For this reason we decided to add human neutrophils to the culture and incubate for 24 h. We then removed the neutrophils from the culture supernatant by centrifugation and the original medium was added back to the epithelial cells for a further 24 h, for a total of 48 h.

### Epithelial cells cultured in neutrophil-conditioned medium and preparation of conditioned media

Again bronchial epithelial cells were cultured in 6- or 12-well plates until 60–70% cell confluence. Cells were then treated with neutrophil-conditioned medium (without neutrophils) to determine whether cell to cell contact was necessary for the induction of EMT. Neutrophil conditioned medium was obtained by culturing neutrophils (0.1-1 × 10^6^/ml) in serum-free RPMI for 24 h. Supernatants were collected, and cells and debris were removed by centrifugation to obtain the conditioned media which was then used for experiments.

### Semi-quantitative RT-PCR

Total RNA was isolated from cultured primary bronchial epithelial cells and purified using the Nucleospin RNA Kit (Takara Bio, Germany) following the manufacturer’s instructions. After extraction, RNA concentrations and purity were measured by spectrophotometry using the Epoch Spectrophotometer System. 200 ng of RNA per sample was then reverse transcribed to cDNA using the Iscript reverse transcription kit (Bio-Rad, CA) and Nexus machine following the supplier’s guidelines. cDNA was stored at -20 °C and remaining RNA at − 80 °C. All primers were purchased from Life Technology (Table 2). Reaction mix without cDNA was used as a negative control, and the human GAPDH gene was used as the housekeeping gene (Table 2). cDNA was subjected to PCR using the SsoAdvanced Universal Sybergreen Green Supermix PCR Master Mix (Bio-Rad) to amplify human transcripts of genes that are markers of the EMT process such as: E-cadherin, N-cadherin, alpha smooth muscle actin and vimentin using their respective sense and antisense primers (Life Technologies) (Table 2). The PCR reaction was carried out in duplicates and according to the manufacturer’s recommended thermal cycling protocol.

**Table 2:** Forward and reverse primers of EMT markers and their oligo sequences

Primer name	Oligo sequence (5′ to 3′)
GAPDH Forward	GAAGGTGAAGGTCGGAGT
GAPDH Reverse	GAAGATGGTGATGGGATTTC
N-Cadherin Forward	CTCCATGTGCCGGATAGC
N-Cadherin Reverse	CGATTTCACCAGAAGCCTCTAC
E-Cadherin Forward	GCCGAGAGCTACACGTTCA
E-Cadherin Reverse	GACCGGTGCAATCTTCAAA
Vimentin Forward	GTTTCCCCTAAACCGCTAGG
Vimentin Reverse	AGCGAGAGTGGCAGAGGA
aSMA Forward	CCGACCGAATGCAGAAGGA
aSMA Reverse	ACAGAGTATTTGCGCTCCGAA

### Immunofluorescence

Some of the isolated neutrophils were used to prepare slides for immunostaining by cytospin. Neutrophils were suspended in RPMI medium at 500,000 cells per millilitre and 80 μL was used per slide. Neutrophils were then fixed with a 4% solution of paraformaldehyde for 20 min, and the slides stored at -20 °C. The Ventana DISCOVERY ULTRA automated slide preparation system was used to stain the neutrophils. The RUO DISCOVERY Universal protocol was applied where the primary antibody specific for human TGF-β1 (MAB-240, R&D Systems) at a 1:100 dilution was incubated for 60 min followed by fluorescently-tagged secondary antibodies (R&D Systems). Cell nuclei were stained with DAPI and the slides were observed with an Olympus immunofluorescence microscope.

### Enzyme-linked immunosorbent assay (ELISA)

ELISA was used to quantify TGF-β1 in neutrophil conditioned medium and culture supernatants. Neutrophil-conditioned media were collected and centrifuged at 300×g for 5 min at 4 °C to eliminate cells and cell debris. Supernatants were then collected and stored at -80 °C. A TGF-β1 ELISA kit purchased from R & D systems (Minneapolis, USA) was used, according the manufacturer’s protocol, to quantify protein amounts in the conditioned media. TGF-β1 protein amounts in unconditioned medium were also measured to account for any small amounts that could be already present in the medium. In preliminary experiments, we measured TGF-β1 with and without activation (adding 1 N HCl) in neutrophils from both asthmatics and non-asthmatics, we found no differences in TGF-β1 levels. This suggests that TGF-β1 was constantly being activated. As such, we believe we were measuring the active form of TGF-β1 in our experiment.

### Analysis of epithelial morphology

Images of live cell cultures were taken using light microscopy. Changes in epithelial cell morphology were then quantified using Image-J’s image analysis tools. Superimposed cells were removed from the analysis as the borders of superimposed cells were very difficult to define. Cell shape measurements (roundness, Feret’s diameter, aspect ratio) were obtained for all the individual cells, as well as an average per image. At least 3 images were analyzed per condition.

### Neutrophil-epithelial cell co-cultures with anti-TGF- β1 neutralizing antibody

Severe asthmatic neutrophils suspended in the co-culture medium were pre-treated with 20 μg/mL of anti-human TGF-β1 antibody (MAB240, R&D Systems) for 2 h. Bronchial epithelial cells were then co-cultured with the neutrophils in the presence of anti-human TGF-β1 neutralizing antibody (20 μg/mL) for 48 h as previously described. Total RNA was isolated and purified to study changes in EMT marker expression.

### Statistical analysis

Standard statistical t-tests and one-way ANOVA were performed to test for statistical significance between data groups.

## Results

### Neutrophils affect the expression of EMT markers when co-cultured with epithelial cells

Peripheral blood neutrophils were efficiently isolated from non-asthmatic and severe asthmatic individuals (purity > 99%) (Table 1). The cell viability was also obtained by trypan blue viability assay, at the time of isolation, and 12 h, 24 h and 48 h post-isolation, the following percentages represent cell viability after these time points respectively: 98, 92, 65 and 34%. Two types of cell culture were performed in this study using the peripheral blood neutrophils from non-asthmatic or severe asthmatic individuals. Neutrophil conditioned medium was obtained and used to treat primary epithelial cell cultures, or neutrophils were directly co-cultured with the epithelial cells.

**Fig. 1:**
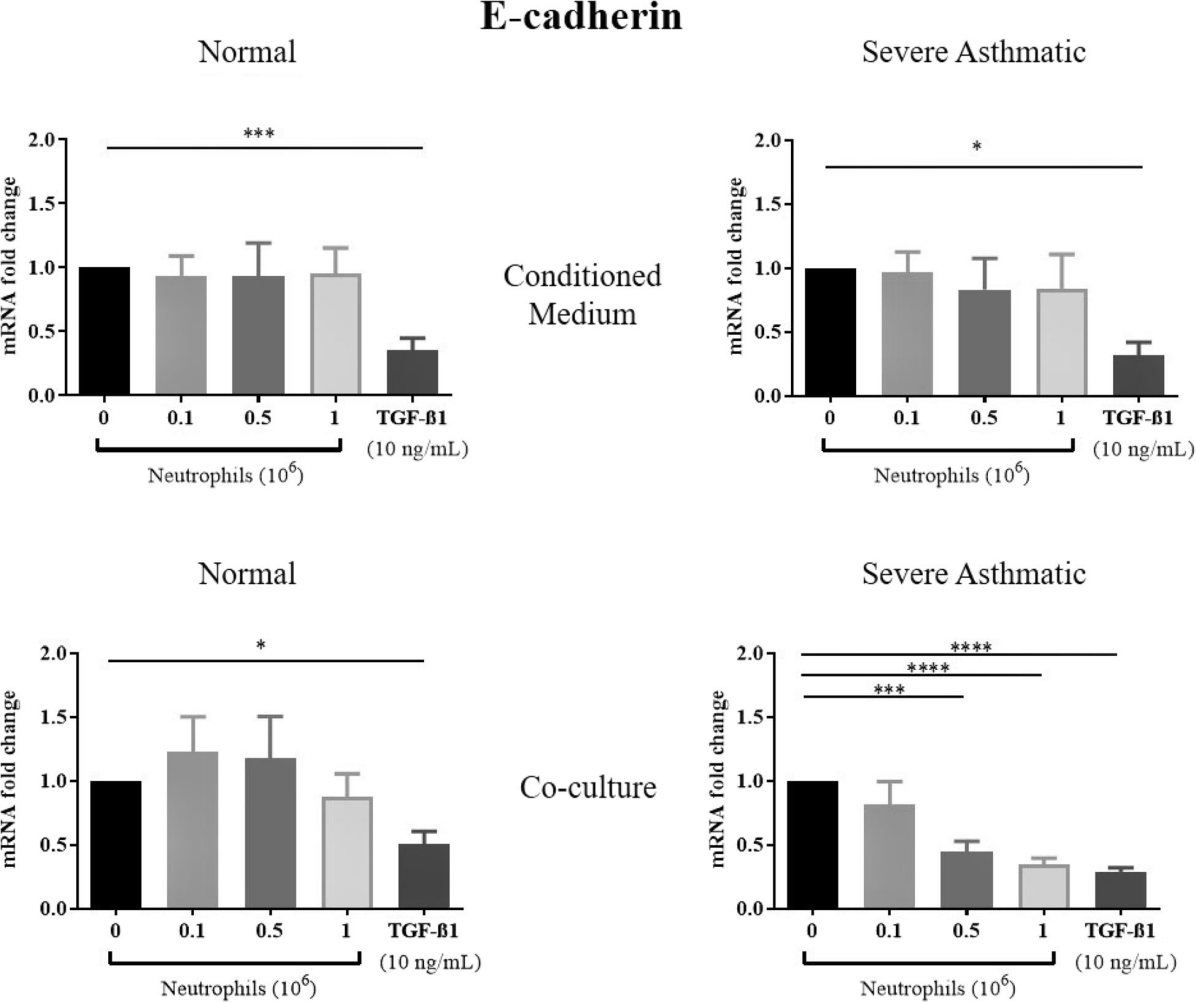
Neutrophils from severe asthmatic patients induce changes in E-cadherin mRNA in human bronchial epithelial cells consistent with EMT. qPCR analysis of E-cadherin mRNA expression of NHBEs following treatment with neutrophil conditioned medium (top panels) or when co-cultured with peripheral blood neutrophils (bottom panels). Neutrophils were obtained from non-asthmatic individuals (left panels) or severe asthmatic individuals (right panels). *N* = 4, Mean ± SE; *P* < 0.05(*), *P* < 0.01(**), *P* < 0.001(***), *P* < 0.0001(****)

**Fig. 2:**
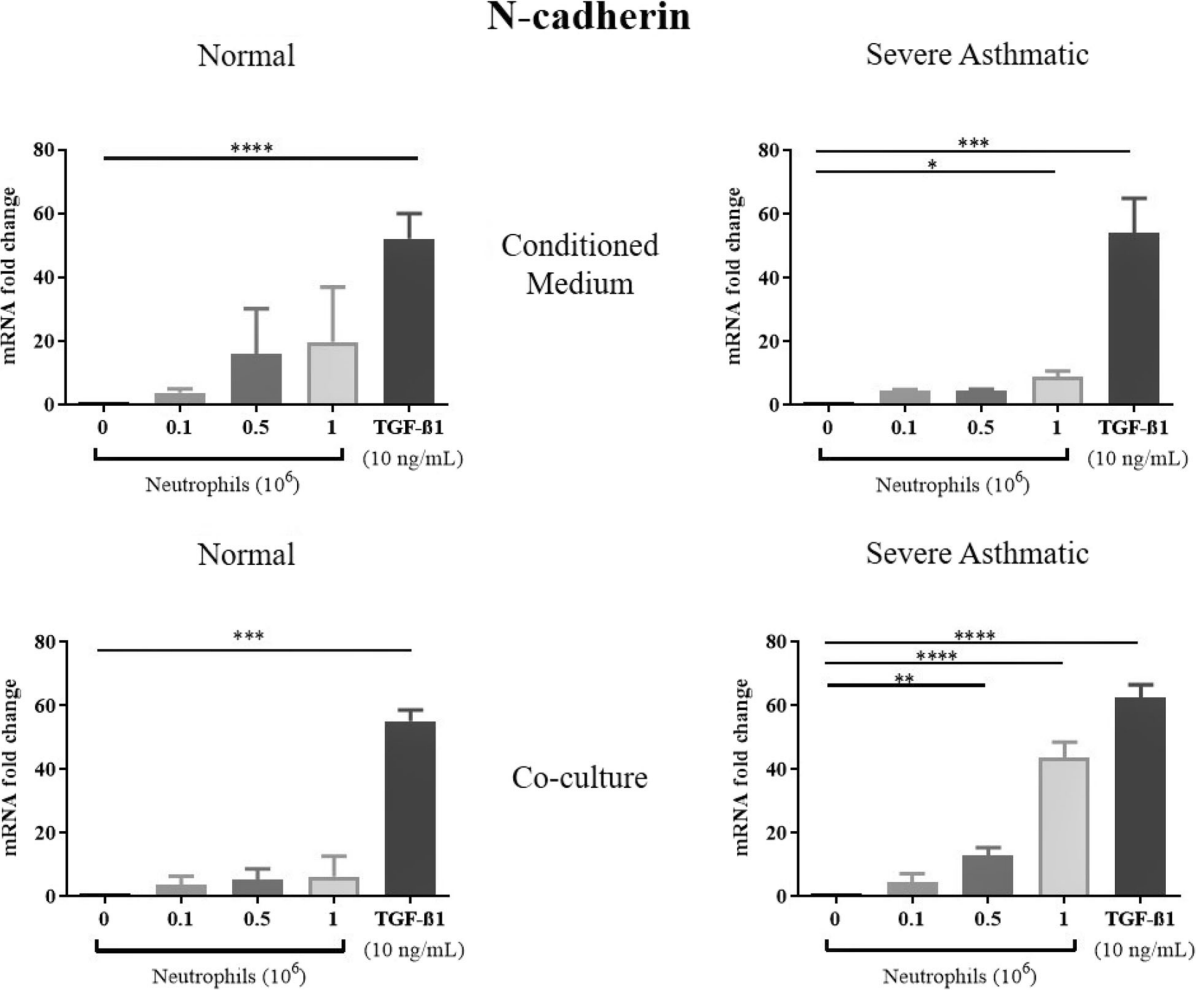
Neutrophils from severe asthmatic patients induce changes in N-cadherin mRNA in human bronchial epithelial cells consistent with EMT. qPCR analysis of N-cadherin mRNA expression of NHBEs following treatment with neutrophil conditioned medium (top panels) or when co-cultured with peripheral blood neutrophils (bottom panels). Neutrophils were obtained from non-asthmatic individuals (left panels) or severe asthmatic individuals (right panels). *N* = 4, Mean ± SE; *P* < 0.05(*), *P* < 0.01(**), *P* < 0.001(***), *P* < 0.0001(****)

**Fig. 3:**
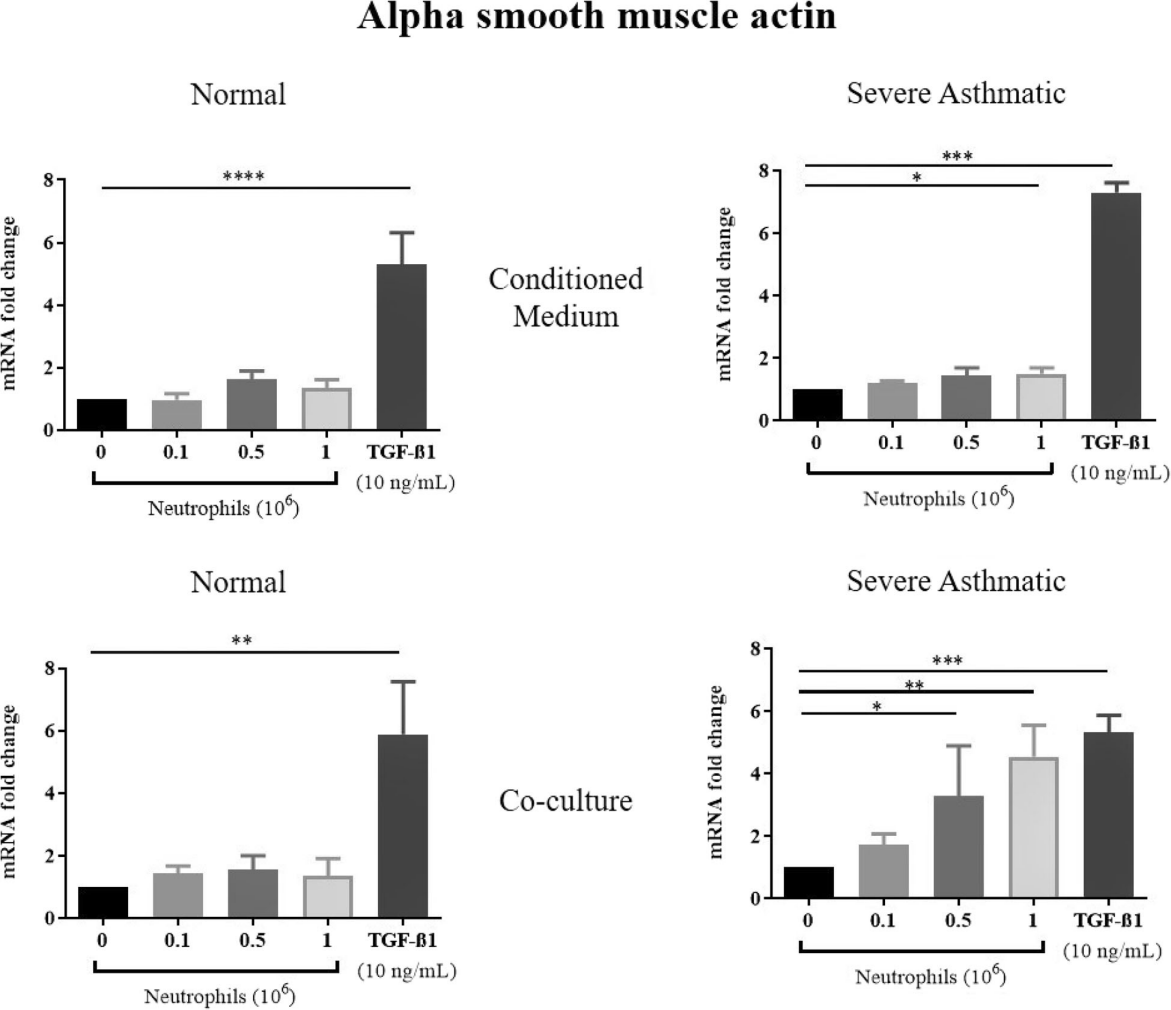
Neutrophils from severe asthmatic patients induce changes in alpha smooth muscle actin mRNA in human bronchial epithelial cells consistent with EMT. qPCR analysis of Alpha smooth muscle actin mRNA expression of NHBEs following treatment with neutrophil conditioned medium (top panels) or when co-cultured with peripheral blood neutrophils (bottom panels). Neutrophils were obtained from non-asthmatic individuals (left panels) or severe asthmatic individuals (right panels). *N* = 4, Mean ± SE; *P* < 0.05(*), *P* < 0.01(**), *P* < 0.001(***), *P* < 0.0001(****)

**Fig. 4:**
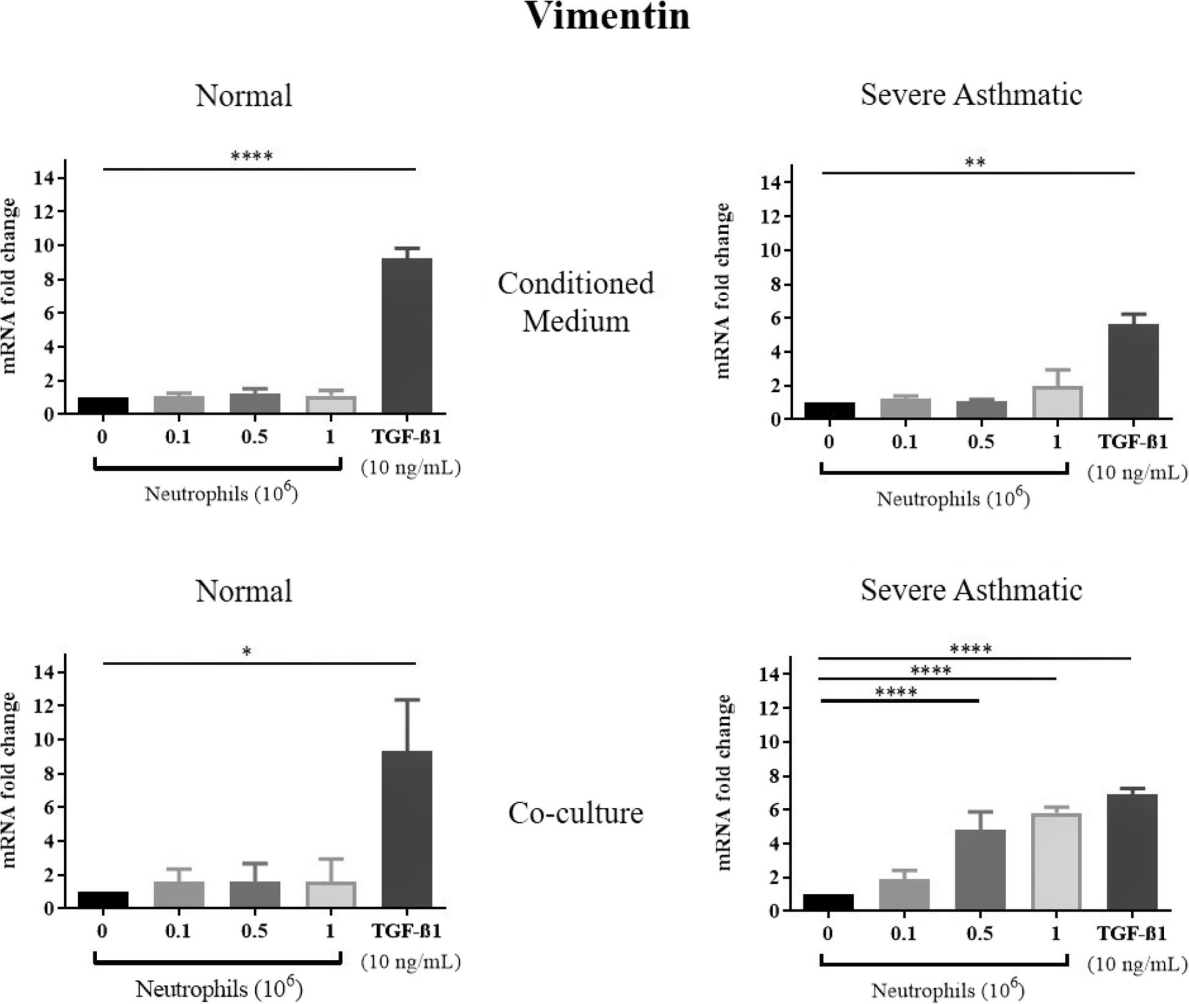
Neutrophils from severe asthmatic patients induce changes in Vimentin mRNA in human bronchial epithelial cells consistent with EMT. qPCR analysis of Vimentin mRNA expression of NHBEs following treatment with neutrophil conditioned medium (top panels) or when co-cultured with peripheral blood neutrophils (bottom panels). Neutrophils were obtained from non-asthmatic individuals (left panels) or severe asthmatic individuals (right panels). *N* = 4, Mean ± SE; *P* < 0.05(*), *P* < 0.01(**), *P* < 0.001(***), *P* < 0.0001(****)

TGF-β1 treatments of NHBE induced EMT, as shown by a significant reduction in mRNA expression of E-cadherin, and increased N-cadherin, αSMA and vimentin mRNA expression (Figs. 1, 2, 3, 4). These results were consistent with previous studies showing that TGF-β1 is a potent EMT inducer. We found that neutrophils from severe asthmatics induced a significant reduction in E-cadherin expression, and increased N-cadherin, αSMA and vimentin expression only when co-cultured with NHBEs, similar to the response induced by TGF-β1 treatment (Figs. 1, 2, 3, 4). These changes are consistent with the EMT process and were not observed when NHBEs were treated with neutrophil conditioned medium (from both non-asthmatic and neutrophils from severe asthmatics). Although neutrophils from non-asthmatics and NHBE co-cultures did not induce significant changes in E-cadherin and vimentin mRNA expression, we did find a statistically significant increase in N-cadherin and alpha smooth muscle actin expression in epithelial cells treated with the culture supernatant of neutrophils from severe asthmatics.

### Epithelial cells undergo morphological changes when co-cultured with neutrophils from severe asthmatic individuals

Epithelial cells undergoing EMT change in morphology. We quantified these morphological changes using three cell shape descriptors. Feret’s diameter represents the total length of the cell and the aspect ratio is a measure of cell elongation (ratio of length to width). Cell roundness was also measured and averaged for each experimental condition.

**Fig. 5:**
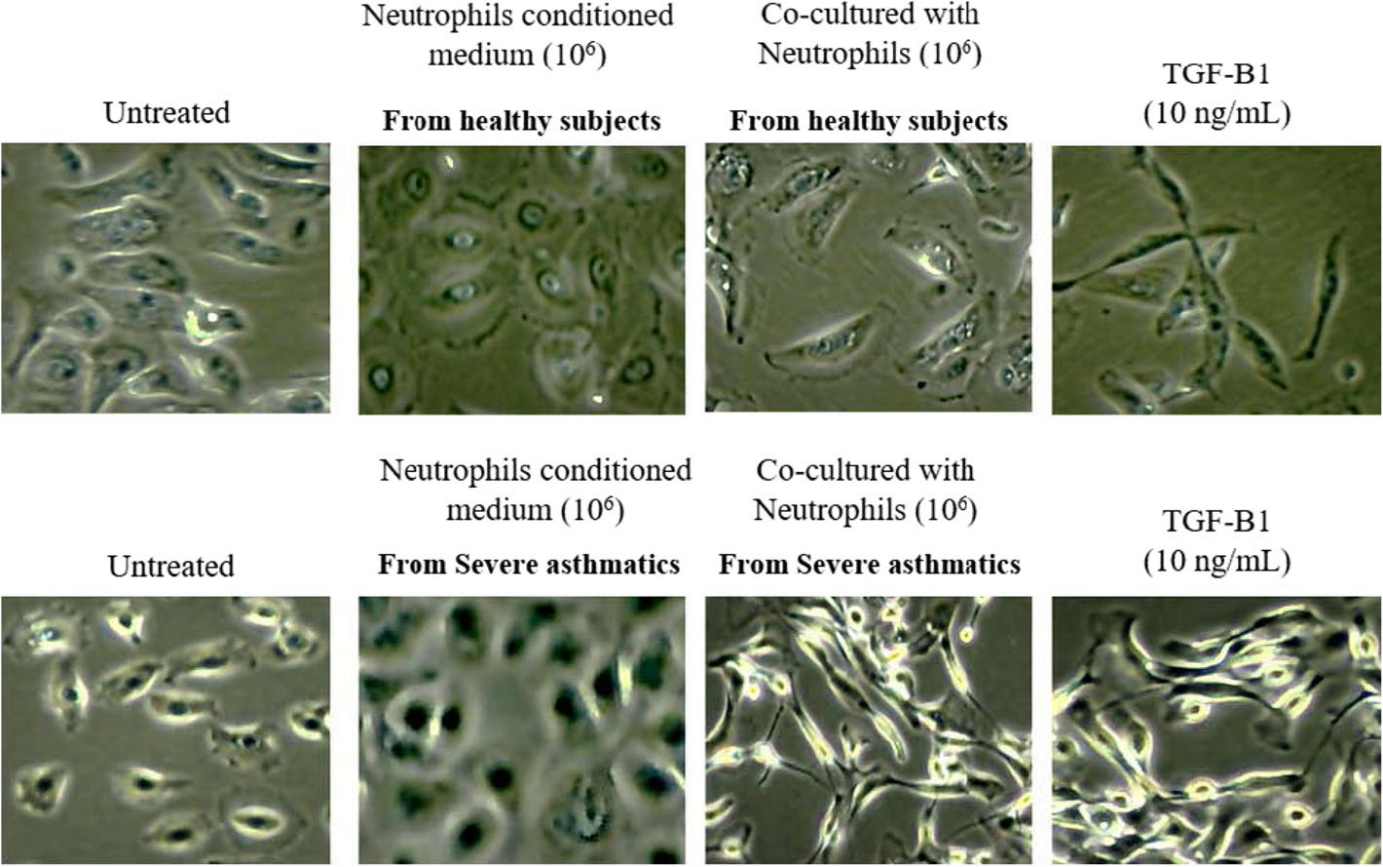
Neutrophils from severe asthmatic patients induce EMT-like morphology in human bronchial epithelial cells. Representative brightfield images of NHBEs untreated, treated with neutrophil conditioned medium, co-cultured with peripheral blood neutrophils or treated with TGF-β1 (left to right) are shown at × 200 magnification (*N* = 4)

**Fig. 6:**
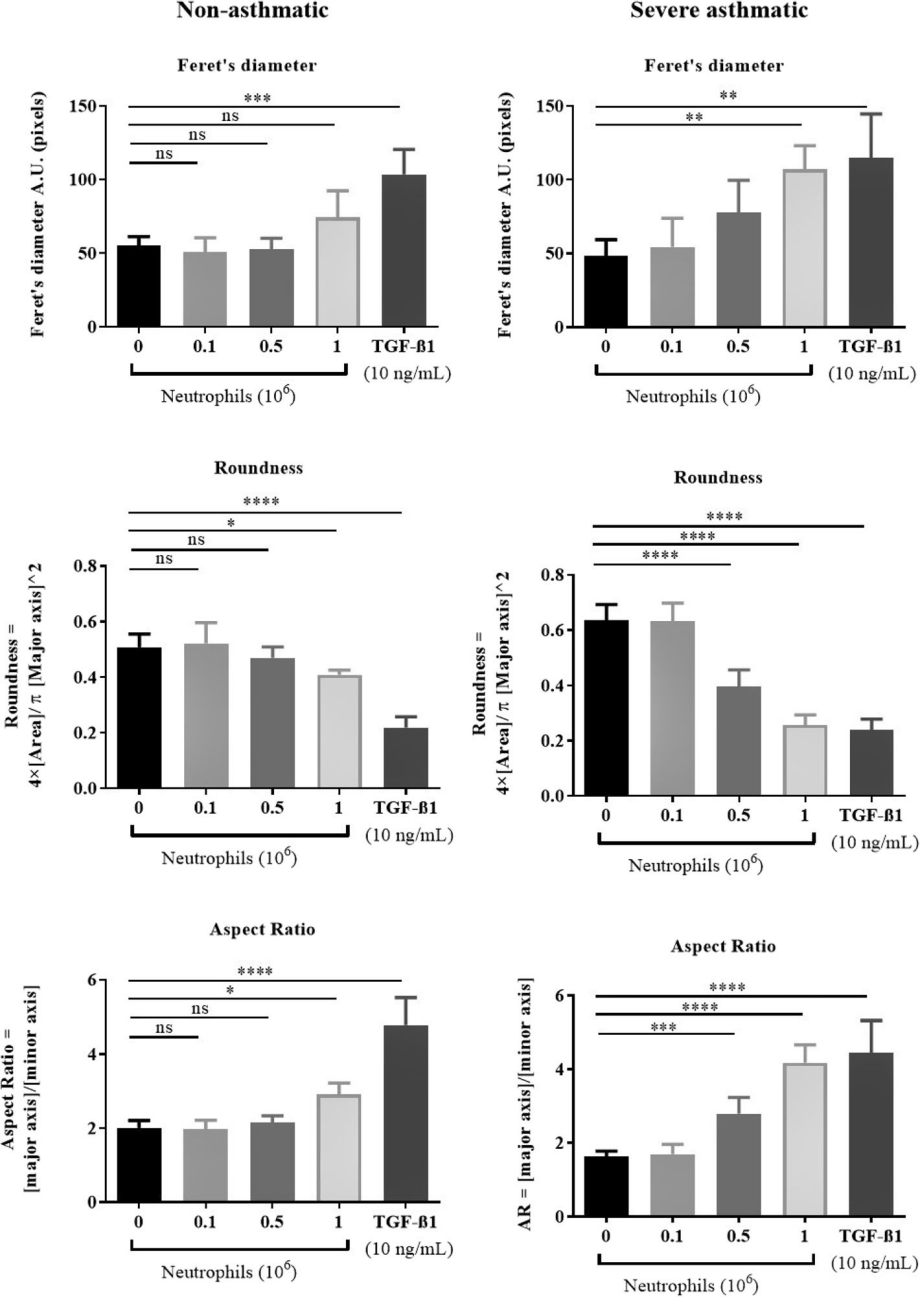
Co-culture of NHBE with neutrophils from severe asthmatics induces a change in epithelial cell morphology. Quantification of epithelial cell shapes following co-culture with non-asthmatic (left panels) or severe asthmatic (right panels) peripheral blood neutrophils. All measurements were performed using Image J’s shape analysis tools from 3 representative live cell culture images per independent experiment. *N* = 4, Mean ± SE; *P* < 0.05(*), *P* < 0.01(**), *P* < 0.001(***), *P* < 0.0001(****)

TGF-β1 treatment of NHBE resulted in significant morphological changes. Both average Feret’s diameter and the average aspect ratio were increased from 48 ± 5.6 to 115 ± 15 (A.U.) and from 1.6 ± 0.051 to 4.4 ± 0.45 (A.U.) respectively, consistent with cell elongation and EMT (Figs. 5, 6). Roundness was significantly decreased upon stimulation with TGF-β1 from 0.5 ± 0.026 to 0.2 ± 0.020 (Fig. 6).

Consistent with our previous results, we found that only NHBEs which were co-cultured with neutrophils from severe asthmatics displayed significant changes in morphology. The average Feret’s diameter and aspect ratio both significantly increased to 107 ± 7.9 and 4.2 ± 0.18 (A.U) respectively, and the roundness decreased to 0.4 ± 0.019 (Fig. 6).

### Neutrophils from severe asthmatic individuals increase TGF-β1 levels in medium when co-cultured with epithelial cells

**Fig. 7:**
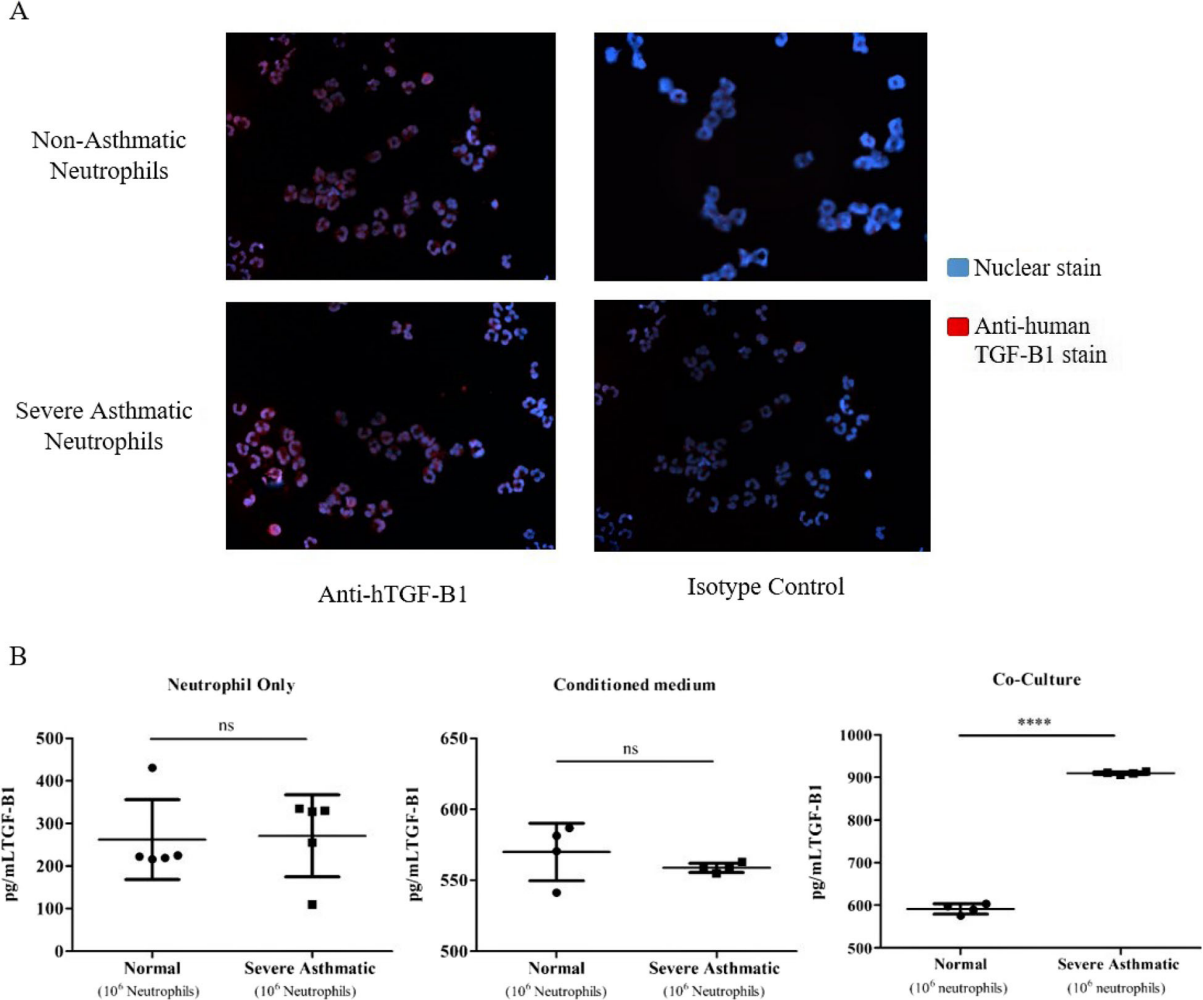
TGF-β1 is secreted by human peripheral blood neutrophils and is increased in neutrophils from severe asthmatics -NHBE co-cultures. **a** Neutrophils from non-asthmatic and severe asthmatic individuals were stained with anti-human TGF-β1 antibodies. TGF-β1 was localized to the cytoplasm. Representative images are shown at 200x. **b** Active TGF-β1 was measured in culture supernatants by enzyme linked immunoscorbant assay. Culture supernatants were collected (from left to right) from untreated cultures of neutrophils (48 h, *N* = 5), NHBEs treated with neutrophil conditioned medium (48 h, *N* = 4) and NHBEs co-cultured with neutrophils (see methods, *N* = 4). Mean ± SE; *P* < 0.0001(****)

TGF-β1 is a pro-fibrotic factor in the airway wall of asthmatics and a potent inducer of EMT and is produced by neutrophils. We performed fluorescent staining of peripheral blood neutrophils using an anti-human TGF-β1 antibody to confirm TGF-β1 expression in these cells. In line with previous reports, we found that normal and neutrophils from severe asthmatics contained TGF-β1 (Fig. 7a).

We then measured TGF-β1 protein levels by ELISA in neutrophil conditioned medium (non-asthmatic vs severe asthmatic) and in culture supernatants (Fig. 7b). We did not find a significant difference in TGF-β1 between normal neutrophil conditioned medium and severe asthmatic neutrophil conditioned medium. In addition, culture supernatant collected following the treatment of NHBEs with neutrophil-conditioned medium did not show any differences in TGF-β1 protein levels when the neutrophils were from severe asthmatic individuals. However, culture supernatant collected from the NHBEs-neutrophils from severe asthmatics co-culture revealed increased amounts of TGF-β1 (910 ± 1.6 pg/mL) compared to supernatant from co-cultures of NHBEs with normal neutrophils (591 ± 6 pg/mL).

### TGF-β1 neutralization reduces the ability of neutrophils to induce EMT

**Fig. 8:**
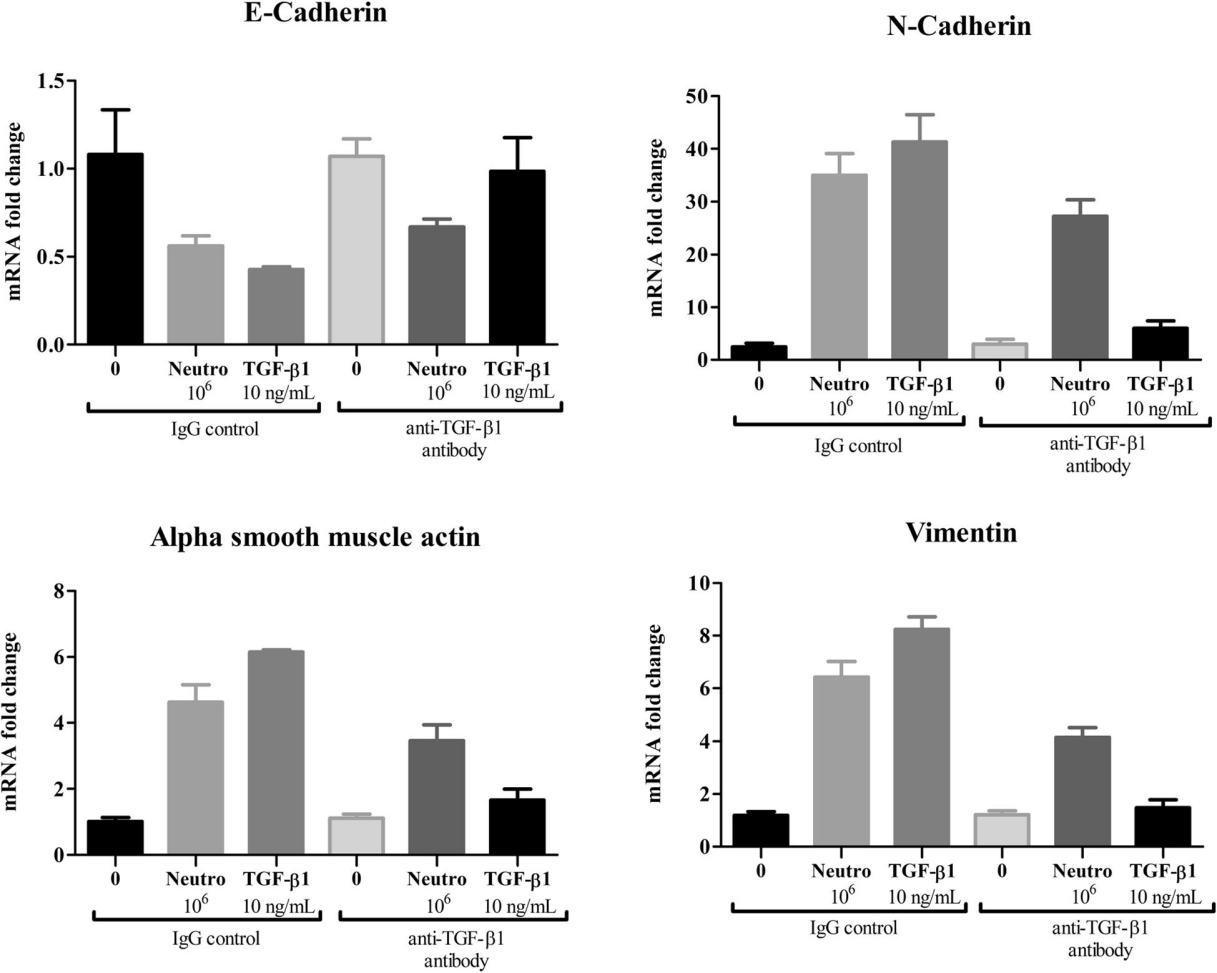
Human anti-TGF- β1 neutralizing antibodies decrease the neutrophil induced expression of EMT markers. Neutrophils from severe asthmatic patients were co-cultured with NHBEs or treated with TGF-β1 in the presence of an IgG control or anti-TGF-β1 antibodies (see methods). The fold changes in mRNA expression of EMT markers are shown. *n*= 2, Mean ± SE

To test whether TGF-β1 was driving EMT in our experiment, we neutralized the cytokine using an anti-human TGF-β1 antibody. We found that neutralizing TGF-β1 reduced the ability of neutrophils from severe asthmatic patients to induce EMT in NHBEs (Fig. 8, *n* = 2). This suggests that TGF-β1 contributes to the induction of EMT, but also that there are other important factors/cytokines involved.

## Discussion

Our results suggest that peripheral blood neutrophils from severe asthmatic subjects have the ability to induce EMT. The changes in EMT marker expression and the acquisition of a spindle shaped morphology observed were consistent with the EMT process. These changes only occurred when epithelial cells were co-cultured with neutrophils or treated with TGF-β1. Therefore, cell-cell contact seems to be required for the induction of EMT by neutrophils. Our data showing the importance of cell-cell contact is similar to those of Yasukawa et al. (40). This group showed that eosinophils are potent EMT inducers using a bronchial epithelial cell line and in vivo. Similarly, they found that EMT induction required direct cell-cell contact. In addition, they reported that eosinophils induce EMT in a TGF-β1 dependent fashion.

In severe asthma patients, eosinophils are major TGF-β1 producing cells (27). However, severe asthma patients with a neutrophil driven inflammation and without lung eosinophilia also may have remodeled airways. Neutrophils can mediate TGF-β1 production in bronchial smooth muscle cells and dendritic cells via neutrophil elastase but are also TGF-β1 producers themselves (24, 25). We confirmed the presence of cytoplasmic TGF-β1 in neutrophils from non-asthmatic and severe asthmatic individuals, as previously reported (39). TGF-β1 is a potent inducer of EMT and a pro-fibrotic factor in the airway wall of asthmatics. It is increased in the airway walls of severe asthmatic individuals (27–30). This led us to consider the role of TGF-β1 in the neutrophil mediated EMT induction in NHBE cells. TGF-β1 was not significantly increased in neutrophil conditioned medium from neutrophils of severe asthmatics compared to neutrophils of non-asthmatic, indicating that the amounts of intrinsically released TGF-β1 by normal and asthmatic neutrophils was similar. Our results contrast with those of Chu et al. (39). Although they reported that the percentage of TGF-β expressing neutrophils was similar between asthmatic and normal airway tissue, they found that peripheral blood neutrophils from asthmatic subjects spontaneously released more TGF-β than those from non-asthmatic subjects. The amount of intrinsically released TGF-β1 could be dependent on the isolation procedure, which can mechanically activate neutrophils. To minimize cell activation, we used an immunomagnetic isolation by negative selection.

The objective of our co-culture experiments was to have neutrophils and NHBE be in contact, allowing for potential crosstalk. Neutrophils have been shown to mediate TGF-β1 production in bronchial smooth muscle cells and dendritic cells and could affect TGF-β1 production in epithelial cells (24, 25). We found that TGF-β1 levels were increased in the culture medium of neutrophils from severe asthmatics -NHBE co-cultures compared to that of neutrophil of non asthmatics-NHBE co-cultures. These findings suggest that neutrophils may be inducing EMT directly via TGF-β1 or by signaling epithelial cells, which are also TGF-β1 producers, to release the cytokine. The autocrine effect of TGF-β1 in the context of EMT has been previously reported (41).

To test whether TGF-β1 was the driver of EMT in our experiment, we neutralized the cytokine using an anti-human TGF-β1 antibody. To account for the possible contribution of epithelial TGF-β1, we pretreated bronchial epithelial cells with the neutralizing antibody before adding neutrophils. Our data, although preliminary, suggest that the EMT inducing ability of severe asthmatic neutrophils is reduced but not completely blocked by neutralizing TGF-β1. This is consistent with the hypothesis that TGF-β1 contributes to the induction of EMT, but that other factors are likely involved.

TGF-β1 is the most potent and well-studied inducer of EMT. However, many other EMT inducers have been described. Those include epidermal growth factor (EGF), fibroblast growth factor (FGF), connective tissue growth factor, insulin-like growth factor-2 (IGF-II), interleukin-1 (IL-1) and hepatocyte growth factor (HGF) (42). Wnt ligands have also been shown to induce EMT. The effect of these mediators can be increased in response to damage to the basement membrane caused, for example, by MMPs. Furthermore, treating epithelial cells with EGF was shown to facilitate survival of cells undergoing EMT (43).

EMT can also occur in response to stresses in the cellular environment. Zhou et al. (44) have shown using an in vitro system, that reactive oxygen species were necessary to induce EMT by hypoxia in transformed and primary human, rat and mouse alveolar epithelial cells. Similar observations were made in renal tubular epithelial cells by Rhyu et al. (26). Neutrophils are known producers of ROS, and the latter could be an important mediator of the neutrophil induced differentiation we have reported. Therefore, it is important to consider the possibility that the induction of EMT observed is a result of a combination of factors and cellular environmental conditions, influenced by both the neutrophils and bronchial epithelial cells.

Our data suggests that TGF-β1 is involved in the neutrophil mediated EMT induction. ROS, MMPs and changes in pH may be other important components of the neutrophil mediated EMT induction mechanism.

## Conclusions

In this study, we showed that peripheral blood neutrophils from severe asthmatic patients can induce the EMT process when co-cultured with primary human bronchial epithelial cells. We also confirmed that neutrophils produce TGF-β1, as previously reported. We did not find significant differences in TGF-β1 secretion between neutrophils from healthy and severe asthmatics. However, we observed an increase in total TGF-β1 amounts in the culture supernatant of severe asthmatic neutrophil-epithelial cell co-cultures, as compared to normal neutrophil-epithelial cell co-cultures. Blocking TGF-β1 modified the process of EMT. These findings suggest that neutrophils contribute to the pathophysiology of asthma by inducing EMT in bronchial epithelial cells. These data support the importance of neutrophils in contributing to airway remodeling in asthma.

## Data Availability

All data generated or analysed during this study are available from the corresponding author on reasonable request.

## Abbreviations

ASM: Airway smooth muscle
BALF: Bronchoalveolar lavage fluid
ECM: Extracellular matrix
EGF: Epidermal growth factor
EMT: Epithelial-Mesenchymal transition
FEV (1): Forced expiratory volume at 1 s
HGF: Hepatocyte growth factor
ICS: Inhaled corticosteroids
MMP: Matrix metalloproteinases
ROS: Reactive oxygen species
TGF-β: Transforming growth factor-beta
TH2: T-helper type 2
α-SMA: A-smooth muscle actin
